# 

**Published:** 1854-05

**Authors:** 


### CONTENTS.

ORIGINAL COMMUNICATIONS:— PAGE

ART. I.—On the Uses of Turpentine. By Samuel Thompson, M. D., 193
ART. II.—Collodion in Erysipelas. By Hiram Nance, M. D., . 196
ART. III.—A Case from my Note Book. By J. H. Hinsey, M. D., 201

SELECTIONS:—

On the Connection of Rheumatism with Scarlatina, . . . 205
On the Treatment of Phthisis by Iodine Inhalations. . . . . 208
Treatment of Psychical Disturbances in their first stage. . . . 212
Chloroform in Obstetrics, . . . . . . . . . 216
Treatment of Diarrhœa by Sulphuric Acid, . . . . . . 216
Common Salt in Intermittent Fever, . . . . . . . . 217
On Insufficient Alimentation, and the Value of Phosphate, &c., . 216
Observations on the use of the Tinct. Ferri. Mur. in Scarlatina, . . 221

EDITORIAL:—

The American Medical Association, . . . . . . . 225

Lardner's Natural Philosophy, etc., . . . . . . . . 237

